# Eco-evolutionary experience and behavioral innovation in interactions with non-native species

**DOI:** 10.1016/j.isci.2024.109462

**Published:** 2024-03-08

**Authors:** Florian Ruland, Andreas A. Meltl, Muriel S. Neugebauer, Jonathan M. Jeschke

**Affiliations:** 1 Leibniz Institute of Freshwater Ecology and Inland Fisheries (IGB), Müggelseedamm 310, 12587 Berlin, Germany; 2 Department of Biology, Chemistry, Pharmacy, Institute of Biology, Freie Universität Berlin, Königin-Luise-Str. 1-3, 14195 Berlin, Germany; 3 Berlin-Brandenburg Institute of Advanced Biodiversity Research (BBIB), Königin-Luise-Str. 2-4, 14195 Berlin, Germany; 4 West Iceland Nature Research Centre (Náttúrustofa Vesturlands), Hafnargata 3, 340 Stykkishólmur, Iceland

**Keywords:** Ecology, Biological sciences, Evolutionary biology

## Abstract

Behavioral changes play an important role for animals to cope with human-induced rapid environmental change such as biological invasions. The concept of eco-evolutionary experience (EEE) postulates that native species are more strongly impacted by non-native species the more these differ from species they have coevolved with. Also, EEE could influence the degree of innovation in new behaviors shown by native species. We conceived categorization schemes to assess both EEE and innovation and applied them to 86 records of behavioral change in native birds (n = 50), mammals (n = 19), and amphibians (n = 17). The results of this proof-of-concept study suggest an interconnectedness of EEE, innovation, and resulting population dynamics of native species. However, quantitative analyses were limited by the small size of our dataset. We encourage the use of the categorization schemes proposed here to close important knowledge gaps, so that our findings can be revisited with larger datasets in the future.

## Highlights

• Native species have a specific eco-evolutionary experience (EEE) with non-native species
• This EEE shapes behavioral changes including innovation
• We conceived and applied categorization schemes to assess both EEE and innovation
• EEE, innovation, and population dynamics of native species appear to be interconnected

## Graphical abstract

**Figure:**
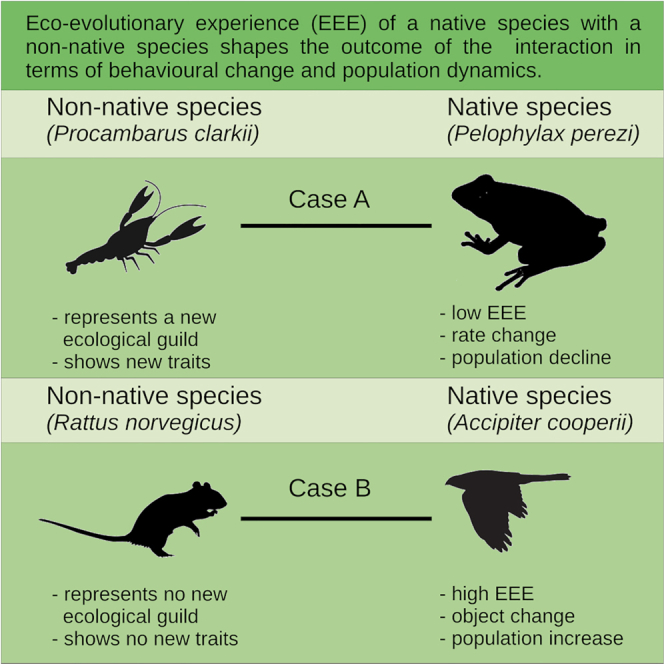

## Introduction

Animal behavior has been recognized to be a crucial mechanism for animal species to cope with all forms of human-induced rapid environmental change (HIREC),^1^ including biological invasions.^2^ A behavioral change is a "first line of defense" of native species coping with non-native species.^3^^,^^4^ At the same time, behavioral flexibility of non-native species can be crucial for their invasion success.^5^^,^^6^ Because such a behavioral change takes place in novel ecological interactions (between the native and non-native species), it can, at least to a certain degree, be considered an innovation sensu Kummer and Goodall^7^: a "solution to a novel problem or a novel solution for a known problem."

Innovation is widespread across the animal kingdom,^8^ but its quantification has remained difficult.^9^ There are detailed methods to look for various forms of innovation in well-known systems with high data ability,^10^ but such procedures are not feasible for other systems. Another approach, which is used in macroecological studies, is focusing on innovation in feeding behavior—the most commonly reported type of behavioral change.^11^^,^^12^ Feeding behavior, however, accounts for less than half of behavioral changes reported in studies on biological invasions.^13^

A change in defense behavior is just as commonly observed in native species and how appropriate it is depends on the species' naïveté with the non-native predator or ecologically similar species.^14^ The impact of non-native species is therefore highest at the initial interaction and decreases with the number of generations of coexistence and the resulting familiarity of the native with the non-native species,^15^ illustrating how rapid evolutionary change can facilitate the appropriate behavioral response.^16^ The ecological consequences of interactions between native and non-native species vary greatly and are of high conservation concern.^17^^,^^18^^,^^19^

The concept of eco-evolutionary experience (henceforth EEE) can help us predict the outcome of novel species interactions based on their shared evolutionary history. On the one hand, EEE can predict invasion success *a priori* by the similarity of native species to those that the non-native species has interacted with in its evolutionary past, and vice versa: a high EEE of the non-native species and low EEE of the native species means that the predicted probability of invasion success is high and negative impacts of the non-native species in its new range are large.^20^^,^^21^ In this case, the invader has a "novelty advantage" (see Jónsson et al.^22^ for a case example of an invader with such a novelty advantage and its long-term impacts). On the other hand, native species can be unaffected by certain non-native species or even benefit from exploiting them as a food source,^23^ which is predicted to be the case if the native species’ EEE is high. Related terms are ecological or functional distinctiveness (a high distinctiveness of non-native species corresponds to a low EEE of native species^24^) and eco-evolutionary naiveté (naiveté of native species is high if EEE is low^15^). The idea of EEE is encapsulated in a number of hypotheses in the field of invasion biology.^20^ However, there is currently no easy-to-use framework to quantify EEE, even though the behavioral response of the native species, its degree of innovation, and population dynamics consequences will depend on EEE.

In this study, we were interested in the relationship between EEE, the degree of innovation in the behavioral change, and the population dynamics of native species interacting with non-native species. For this purpose, we developed two straightforward classification schemes to assess EEE and the degree of innovation. As a proof-of-concept analysis, we applied these schemes for records of behavioral change in native mammals, birds, and amphibians interacting with non-native species from a recent study.^13^ Specifically, we investigated biases in the types of behavior that were reported to change and assessed the levels of EEE and behavioral innovation for the three taxonomic groups. Further, we explored which behavioral responses were observed for which levels of EEE. Finally, we compared the post-interaction population trends for the native species depending on its behavioral change for different levels of EEE. All analyses were performed for (1) the full dataset; (2) a dataset correcting for phylogenetic autocorrelation where each genus of non-native species was only used once; (3) a subset of native bird species, the most common taxon, to have a taxonomically more homogeneous dataset; (4) a subset of non-native predators; and (5) a subset of non-native prey species.

We addressed the following three hypotheses: hypothesis 1 (H1): there is a negative relationship between EEE and innovation, as non-native species that are similar to the original ecological environment of the native species do not necessitate a drastic change in the native species' behavior. H2: highly innovative behavior should lead to a more positive outcome of the interaction for the native species in terms of population trend compared with less innovative behavior. In other words, there should be a positive correlation between the degree of innovation and population trend. Innovation is predicted to help the species by, for example, evading a novel predator or exploiting a new food source efficiently. H3: similarly, a higher level of EEE of the native species interacting with the non-native species should lead to a more positive outcome in terms of population trend. This is because non-native species that are similar to species the focal native species previously interacted with should be less of a threat as a competitor or predator and more readily ingested as prey.

## Results

Accuracy of classifying EEE was good, with the two researchers (MSN and AAM) initially answering the questions on the same guild, a new trait, and a new functional trait with 75.6%, 73.3%, and 72.1% consistency, respectively. The accuracy of classifying innovation was even better, with consistent initial answers of the two researchers in 83.7%, 91.9%, and 100% of the records for the questions on rate change, object change, or technical change, respectively.

**Figure 1:**
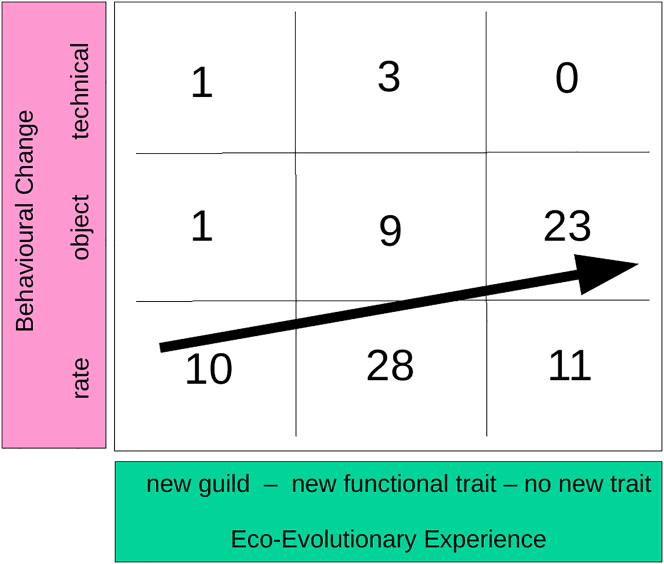
EEE and behavioral change Records of a new guild and/or a new functional trait in the non-native species and the type of behavioral shift as the native species' response (rate, object, or technical change). The level of eco-evolutionary experience (EEE) increases from left to right and the level of innovation from the bottom to the top. The arrow denotes a trend toward more object innovations with increasing EEE. Total n = 86 records.

In the EEE assessments, we found for 12 of the 86 records in our dataset that the non-native species represented a guild unknown to the native species (Figure 1, left column). One example is the interaction between red swamp crayfish (*Procambarus clarkii*) and Iberian water frogs (*Pelophylax perezi*) in Portugal in a system where crayfish were not previously present^25^; another example is the predation of lesser sheathbills (*Chionis minor*) by invasive mice (*Mus musculus*) in Marion Island where no rodent predators were originally present.^26^ Invasive species were observed to display a new behavioral trait in more than half of the records (n = 50), and it was almost always a new functional trait (n = 47, Figure 1, 40 cases in the central column and 7 in the left). We therefore decided to limit our subsequent analyses to new functional traits. There were 34 cases of non-native species being from a known guild and not showing new functional traits (Figure 1 right column).

Native species frequently responded with a rate change (28 out of 40 records) to non-native species with a new functional trait. For non-native species without a new functional trait, the native species more commonly performed an object innovation, directly interacting with the non-native species (23 out of 34 records, Figure 1, p < 0.001, X^2^ = 18.9). This is opposite to what we expected and formulated in H1: EEE and innovation were hypothesized to be positively correlated overall. However, when we focus on the four records of technical innovation—the highest form of innovation in our dataset (upper row in Figure 1)—we find that they occurred for low levels of EEE, either in interactions with non-native species from a new guild or in interactions with a new functional trait. This finding is in line with H1, although the sample size is small.

The innovation assessments revealed that after the onset of the interaction, most native species changed the rate of the focal behavior in their repertoire (Figure 1, bottom row). Many native species also changed the object of interaction, with the new object always being the non-native species in our dataset. There were four records of technical change, and all of them coincided with an object innovation, i.e., with the non-native species as the new object of interest: the black-capped chickadee (*Poecile atricapillus*) in western Montana, which showed a hovering technique to exploit a novel food source^27^; the same study reports this behavior in mountain chickadees (*Poecile gambeli*). Another example comes from the long-fingered bat (*Myotis capaccinii*) that feeds on invasive mosquitofish (*Gambusia affinis*) in North-Western Israel.^28^ These fish do not only represent a novel food item, but the long-fingered bat is the first bat species in the middle East for which piscivory has been shown. Finally, new world warblers (*Geothlypis trichas*) were shown to feed on the complex flowers of *Strelitza reginae* (originally from South Africa) in southern California, thereby pollinating them.

**Figure 2:**
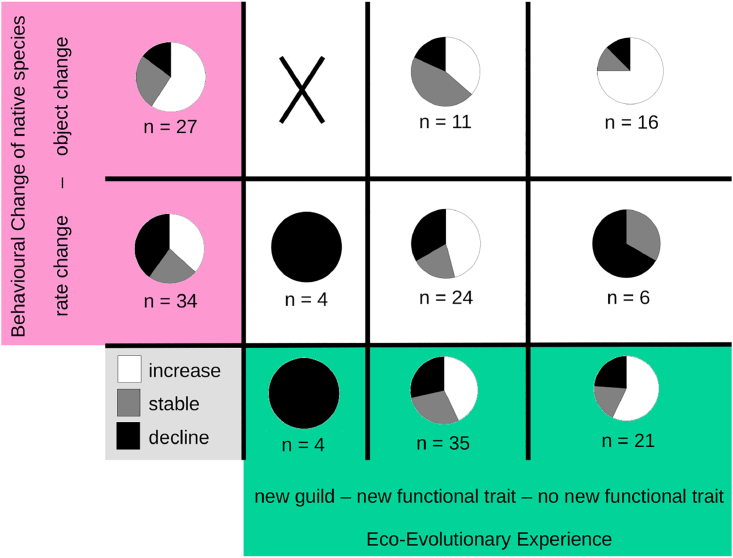
EEE, behavioral change and population trends All records with population trends—post-invasion population trends of the focal native species (decline, stable, or increase in the pie charts) depending on their eco-evolutionary experience (new guild or new functional trait, green boxes) and the degree of innovation in their behavioral change (rate change or object change, outcome in magenta boxes).

We were able to analyze 61 records of behavioral change with population trend data of the native species after the onset of its interaction with the non-native species (Figure 2). We found generally supportive evidence for H2. In particular, there was a significant difference between population trends after rate and object changes, respectively (p < 0.05, X^2^ = 7.4), with object changes leading to more positive population dynamics. This seems to be driven by the interaction of the non-native species’ trait and the native species’ response. In case of the non-native species not showing a new functional trait, native species performing an object change (thus directly interacting with the non-native species) more frequently had a positive population trend than native species performing a rate change (p < 0.01, X^2^ = 10.2). Object change or rate change did not lead to significantly different population trends if the non-native species showed a new functional trait (p = 0.4, X^2^ = 2.3). Finally, for only three records of technical innovations did we have population trend data, which were inconclusive: one native species declined after the technical innovation (*Myotics cappacini*), whereas two species (*Poecile atricapillus* and *P. gambeli*) showed a stable population.

Evidence for H3 was supportive overall, as populations of native species interacting with non-native species from a new guild always declined (p < 0.05, X^2^ = 8.8), followed by interactions with non-native species with a new functional trait (decline in 29% of the records) and without such a trait (decline in 24% of the records), with the latter two not being significantly different, though (p = 0.6, X^2^ = 1.1). Results were qualitatively similar when using each genus only once or only using cases of native bird species (see analyses in Supplement S1).

**Figure 3:**
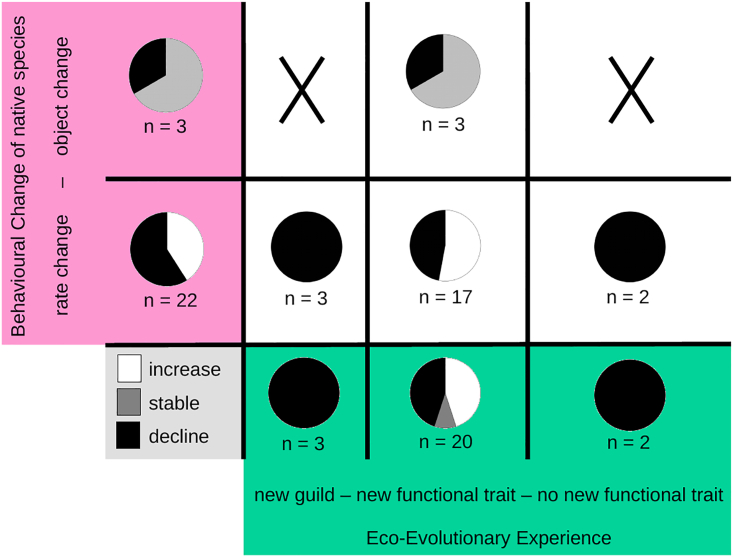
EEE, behavioral change, and population trends of native species in interaction with non-native predators Interactions with non-native predators—post-invasion population trends of the focal native species (decline, stable, or increase in the pie charts) depending on their eco-evolutionary experience (new guild or new functional trait, green boxes) with a non-native predator and the degree of innovation in their behavioral change (rate change or object change, outcome in magenta boxes).

EEE (p < 0.001, X^2^ = 15.4), innovation (p < 0.001, X^2^ = 31.8), and population trend (p < 0.01, X^2^ = 13.2) all were more positive when non-native species were prey for the native species as compared with predator species. However, results were qualitatively similar when performed within the subsets: EEE and object change also were predictors for positive population trend (p < 0.1, X^2^ = 5.3 and p < 0.05, X^2^ = 8.9, respectively; see Figure 3). Notably, no non-native prey species came from a guild unknown to the native species.

For cases of behavioral change in response to invasive predators, overall EEE (p < 0.001, X^2^ = 14.2) and innovation (p < 0.001, X^2^ = 17.1) were lower and population trends more commonly negative (p < 0.01, X^2^ = 11.1). Object changes were less often followed by a negative and never a positive population trend but most commonly stable (p < 0.001, X^2^ = 16.2). EEE did not have a significant effect on population trend (new guild: p = 0.42, X^2^ = 2.7; new functional trait: p = 0.3, X^2^ = 3.2; no new functional trait: p = 0.58, X^2^ = 1.7).

## Discussion

In this study, we developed two general and easy-to-apply schemes to assess (1) the level of EEE in interactions between native and non-native species and (2) the level of innovation in behavioral changes. We applied these schemes to records of behavioral change in birds, mammals, and amphibians interacting with non-native species and also investigated to which degree these interactions affected the focal native species’ population dynamics. Our findings partly supported the three hypotheses stated in the Introduction, which predicted (H1) a negative relationship between innovation and EEE, (H2) a positive relationship between innovation and population trend, and (H3) a positive relationship between EEE and population trend. Due to the small size of our dataset, which led to limitations in statistical analyses, the results reported here should be interpreted cautiously and revisited in future analyses based on larger sample sizes. The current article should mainly be seen as a proof-of-concept study, outlining the two schemes and their application.

### Relationships between EEE, innovation, and population trends

The relationship between technical innovation—the highest form of innovation in our dataset—and EEE supported our H1, according to which low EEE requires more innovative behavior. The native species seem to match the degree of innovation in their behavioral change to the degree of novelty in the non-native species (from the native species’ perspective, cf. Heger et al.^29^). However, we found that this behavioral plasticity is often not sufficient to buffer adverse invader impacts (see also Wong and Candolin^30^). Contradictory to our H2, technical innovations were shown by native species with rather negative population trends: no record of a population increase, two stable records, and one record of a population decline. This is, of course, a very small sample size and does not provide clear evidence against the established idea that innovation buffers against population decline, which was recently supported by a large study on feeding innovations in birds.^31^

The observation that object innovations—in contrast to mere rate changes—occur significantly more often in ecological interactions with high EEE is contradictory to H1. This hypothesis is based on the idea that native species are driven to innovate in novel ecological settings (again, from their perspective sensu Heger et al.^29^). What is not considered in this hypothesis, however, is the benefit of interacting with a non-native species where EEE is high. In these cases, object innovations—directly interacting with the non-native species—actually led to a more positive population trend for the native species, in support of H2. We thereby see the benefit in disentangling the different kinds of innovation and look at them in their specific context as opposed to a general score. We come back to this point in the next section.

Overall, we found more positive population trends for native species with high EEE, in support of H3 (see also Saul and Jeschke^21^). In particular, populations of native species interacting with non-native species from a new guild always declined. Unfortunately, though, we only had four records of behavioral change for which the relevant data were available.

### Animal innovation is context dependent

**Figure 4:**
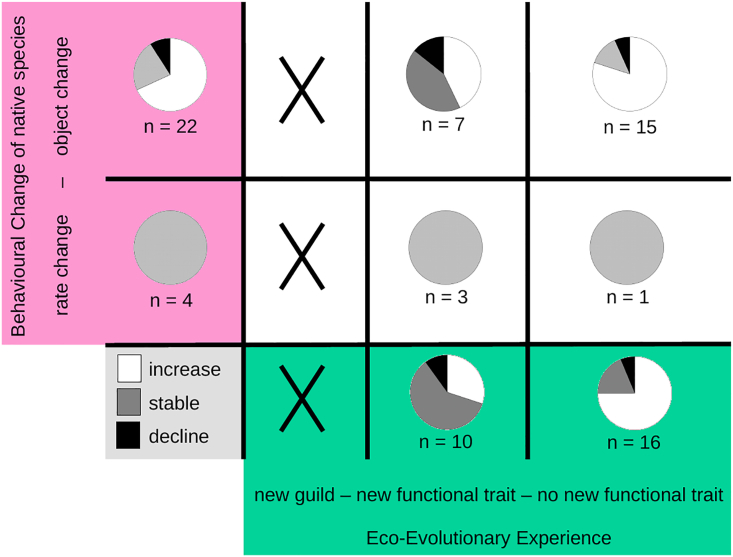
EEE, behavioral change, and population trends of native species in interaction with non-native prey Interactions with non-native prey—post-invasion population trends of the focal native species (decline, stable, or increase in the pie charts) depending on their eco-evolutionary experience (new guild or new functional trait, green boxes) with a non-native prey species and the degree of innovation in their behavioral change (rate change or object change, outcome in magenta boxes).

Although we found generally similar results when focusing on records for birds (Figure S1.4), changes in response to non-native predators (Figure 3) or prey species (Figure 4), comparing the two latter subsets of records is interesting. If the non-native species is a potential prey species, the native species can choose to either interact with (i.e., feed on) the non-native species or not. This difference between feeding and defense behavior has been captured in the life-dinner principle, which posits that adaptations to avoid predators should occur faster than those to capture prey, as consequences of failing to escape from a predator are greater than those of missing an opportunity to eat.^32^ Indeed, we found more records in our dataset of native species feeding on non-native species without a new functional trait (where EEE is high and most native species increased in their population) than with a new functional trait (where EEE is lower and only a minority of the native species increased in their population). One can, of course, imagine ecological settings where an invasive prey species is just too common to ignore or has displaced the preferred prey of the native species and is therefore almost required to be "chosen" as new prey. In such cases, the pressure to innovate in order to successfully attack and feed on the non-native species can be similar to records of changing defense behavior.

In the subset of records with changes in response to non-native predators, we saw few changes for interactions with non-native species without a new functional trait (i.e., the opposite pattern than for changes in feeding behavior). Native species might just not have to change their behavior in these cases: their defense mechanisms are intact, the non-native species does not pose a novel threat, and therefore pressure to innovate is low. If the non-native species does show a new functional trait, however, rate changes more often leads to negative population dynamics than object changes. This aligns with theory that predicts this effect to be driven by native prey failing to defend themselves against a non-native predator they are naive to^33^; a rate change in an existing behavior might just not be sufficient to buffer adverse effects in such cases. These findings, which are based on 25 records of behavioral change across 3 taxonomic groups, hint toward fundamentally different patterns of innovation consequences for different types of behavior.

### Tools for assessing EEE and innovation

Animal innovation can be observed and documented in a wide range of contexts, either when researchers actively looked for it and then highlighted it in a publication or in studies with a different focus. However, we draw most of our knowledge on animal innovation from comparative studies on birds^34^^,^^35^ or primates.^36^^,^^37^ Some of these patterns might be universal, but it is important to also look for innovation outside of these taxonomic groups and achieve a more balanced perspective. Similarly, most large comparative analyses of animal innovation focus on feeding behavior (e.g., Ducatez et al.^31^), whereas other types of behavior are understudied. Our easy-to-use scheme for assessing innovation is applicable across taxonomic groups and types of behavior. It also achieved a high level of consistency between assessors in our study, and the approach outlined in the STAR Methods section allows to reach consensus in cases where assessors disagree. We hope the scheme will be used to assess studies of innovation for other taxonomic groups (e.g., insects) and other types of behavior than “the usual suspects,” so that the current bias in the literature can be balanced out.

Other methods of assessing EEE have been proposed in previous studies. In particular, Saul et al.^20^ outlined a relatively complex calculation scheme based on the Bray-Curtis similarity index. Such a scheme can be used for systems where detailed knowledge is available, but it will not be possible to gather such data for a large number of records, which are for instance needed to address macroecological questions. Another option to assess EEE is to look at phylogenetic relationships: if a non-native species is phylogenetically closely related to species that are already present in the community it was introduced to, one can assume the native species to have a high level of EEE with the non-native species, and vice versa, one can assume a low level of EEE if the non-native species is phylogenetically distant from species that are already present in the community. As outlined by Saul et al.^20^ (p. 62), however: “similarity – be it in respect to morphological, behavioral, or ecological traits – does not necessarily correlate with relatedness.^38^ This becomes most evident in cases of convergent evolution where relatively unrelated species show a high degree of similarity” (see Saul et al.^20^ for references).

### Limitations of the study

This study is limited in data availability. Three taxa were included: several categories of eco-evolutionary experience and behavioral innovation, population trends, and types of interactions (i.e., predation) between native and invasive species. The available 60 records of interactions were split between these categories, and factors were analyzed separately. Overall, the possibilities for statistical testing were impaired by lack of data.

### Outlook

We here focused on biological invasions, but one can easily use the two schemes for assessing EEE and innovation in a different field, for example other instances of human-induced rapid environmental change (HIREC) like urbanisation,^39^ artificial light at night,^40^ or climate change.^41^ Of course, questions concerning EEE or innovation can be added or revised to adapt the schemes for specific ecological settings. We do not suggest a distinctly more complicated catalog of questions as a way forward for comparative studies. It would require very detailed knowledge of the interacting species, which will often be impossible to obtain, e.g., for rare or understudied species. Our approach was to design ready-to-use and straightforward frameworks that still have predictive power in terms of how a non-native species will affect a native species. We encourage the use of both frameworks in future studies to achieve a broader coverage of comparative information on animal innovation and EEE, as the present study was clearly limited in sample size, thus simultaneously controlling for different factors in a single model was not possible. This will be a task for future studies based on a larger sample size. The schemes presented here can in the future perhaps also help predict the outcomes of interactions between native and non-native species and thus serve conservation efforts. For this application, comparisons between EEE and impact scores (based on EICAT or other impact assessment schemes)^42^^,^^43^^,^^44^^,^^45^ will also be very useful.

## STAR★Methods

### Key resources table

**Table:** 

REAGENT or RESOURCE	SOURCE	IDENTIFIER
**Software and algorithms**		

R software version 4.0.1	R development core team	https://www.r-project.org/

**Deposited data**		

Raw data	This paper	https://doi.org/10.5061/dryad.m37pvmd92

### Resource availability

#### Lead contact

Further information and requests for resources should be directed to and will be fulfilled by the lead contact, Florian Ruland (florian.ruland@posteo.de).

#### Materials availability

This study did not generate new unique reagents.

#### Data and code availability

• All data have been deposited at Dryad and are publicly available as of the date of publication. The DOI is listed in the key resources table.
• The paper does not contain original code.
• Any additional information required to reanalyze the data reported in this paper is available from the lead contact upon request.

### Method details

All records of behavioral change come from a systematic literature review of the Web of Science database. The initial search was conducted on 30 June 2015 and subsequent assessment for eligibility yielded 360 records of behavioral changes of either native or non-native species during species invasions coming from 191 studies (Ruland and Jeschke,^13^ see Supplement S1 for details). As we were interested only in the effects of invasions on the population dynamics of native species and their behavioral response, we restricted our dataset to native species changing their behavior. We chose to focus on three vertebrate groups: birds (n = 50), mammals (n = 19) and amphibians (n = 17), which are also common taxa for innovation research in non-human animals. To correct for the overrepresentation of a few closely related species, we repeated all analyses with a subsample of the data where each genus was only used once (n = 39). All records of behavioral change were also split by type of interaction (invasive prey: n = 26, invasive predator: n = 25).

For all records, we checked if the specific native species population under study was increasing, stable or declining after the arrival of the non-native species. This information was – if available – taken directly from statements in the original publications describing the behavioral change. If such information was not provided, the authors of the study were contacted who are in many cases the most knowledgeable experts on the system. We also checked available information on the IUCN Red List of Threatened Species (IUCN^46^) and the Encyclopedia of Life (EOL^47^). We only used the population trend data if we found a concrete statement about how the specific population of native species was affected by the presence of the respective non-native species in the interaction.

#### Eco-evolutionary experience

We assessed the level of eco-evolutionary experience (EEE) of the native species interacting with non-native species by means of three guiding questions, which we developed based on the key literature about this topic (see above for references). First, does the non-native species belong to a new guild (i.e., an ecological guild that was not previously represented in the community)? We followed Root^48^ in defining an ecological guild as "a group of species that exploit the same class of environmental resources in a similar way". Second, we asked: Does the non-native species show a new trait unknown to the native species? The final question was: Is this new trait relevant in the interaction with the native species (i.e., is it a *new functional trait*)?

Let us look at an example to illustrate how these questions can be answered and what consequences this has for the assigned level of EEE for the native species: The invasive red swamp crayfish (*Procambarus clarkii*) is a new predator of the Iberian waterfrog (*Pelophylax perezi*) in Portugal in a system where crayfish were not previously present^25^ – it therefore represents a new guild. Furthermore, the nocturnal feeding pattern of the crayfish is a new functional trait in the system, thus both tadpole and adult stage waterfrogs will have to adapt to crayfish predation at night. This is therefore an example where the native species has a low level of EEE.

#### Behavioral innovation

To classify the degree of innovation in the behavioral response of the native species, we also developed three guiding questions. The first question was: Did the rate of an existing behavior change? Such a rate change represents the lowest degree of innovation; strictly speaking, it is not yet a true innovation. For other cases, where not only the rate of a known behavior changed but also a new behavior was observed, we used the definition by Kummer and Goodall^7^ already mentioned in the Introduction, that innovation is a "solution to a novel problem or a novel solution for a known problem"; we formulated one question for each of its two parts: a "solution to a novel problem" (object change) and a "novel solution for a known problem" (technical change). Hence, our second question was: Does the native species perform a known behavior in interactions with a new object of interest (*object innovation*)? And the third question: Does the native species perform a new action pattern (*technical innovation*)?

An example is the rate change observed in the native California red-legged frog (*Rana draytonii*), which spends more time hidden under branches of willows when the invasive bullfrog (*Lithobates catesbeianus*) is present.^49^ The black-capped chickadee (*Poecile atricapillus*) in western Montana^27^ shows both object and technical innovation: *Urophora* larvae are an exotic pest control and are now commonly found in open habitat on the invasive spotted knapweed (*Centaurea stoebe*). These larvae can be accessed by a novel hovering technique that black-capped chickadees developed and that minimizes their time spent in the exposed and, therefore, risky habitat. Thus, this record of behavioral change represents the highest degree of innovation in our framework.

All questions regarding EEE and behavioral innovation were first independently answered for each record of behavioral change by two assessors (AAM and MSN). Following a Delphi consensus approach,^50^ we then calculated the % consistency in the responses to each question between both assessors. In the third step, the results were exchanged between both assessors, and each of them reassessed and possibly changed their answers for the records in which the answers differed. Fourth, the revised answers were compared to identify records that were still classified differently by the two assessors. Finally, there was a joint discussion between both assessors and a moderator (FR) to reach a consensus for each of these remaining records. Following this approach, we were able to reach consensus in all answers concerning EEE and behavioral innovation for all records of behavioral change analyzed in this study.

### Quantification and statistical analyses

We analyzed the distribution of records over the degrees of EEE and innovation, and then how these are connected to more or less decreasing, stable or increasing native species populations after the onset of the interaction. We compared, for example, the distribution of population trends of native species that interact with non-native species from a new guild vs. the case when the non-native species is not from a new guild or whether rate or object innovation led to a more positive population trend. These analyses were performed for (i) the full dataset and repeated for the three subsets of the data to detect any differences and biases: (ii) each genus only counted once, (iii) birds only, (iv) invasive prey species and (v) invasive predators. We performed Chi-square tests in the open software R,^51^ using the function chisq.test with 100′000 simulations.

## Supplemental information

Document S1. Figures S1.1–S1.4 and Table S1

Document S2. Article plus supplemental information
