# Supplementary material for: Eco-evolutionary experience and behavioral innovation in interactions with non-native species
Source: iScience. 2024 Mar 8;27(4):109462. doi: 10.1016/j.isci.2024.109462 (PMC10973602; doi:10.1016/j.isci.2024.109462)
Supplement: Document S1. Figures S1.1–S1.4 and Table S1 [file mmc1.pdf]

iScience, Volume *27*

Supplemental information

Eco-evolutionary experience and behavioral innovation in interactions with non-native species

Florian Ruland, Andreas A. Meltl, Muriel S. Neugebauer, and Jonathan M. Jeschke

Eco-Evolutionary Experience and Behavioural Innovation in Interactions with Non-Native Species – Supplement S1

Literature search

A flow chart showing the number of studies found in our initial search and how many records remained after each scanning for eligibility is shown in Fig. S1.

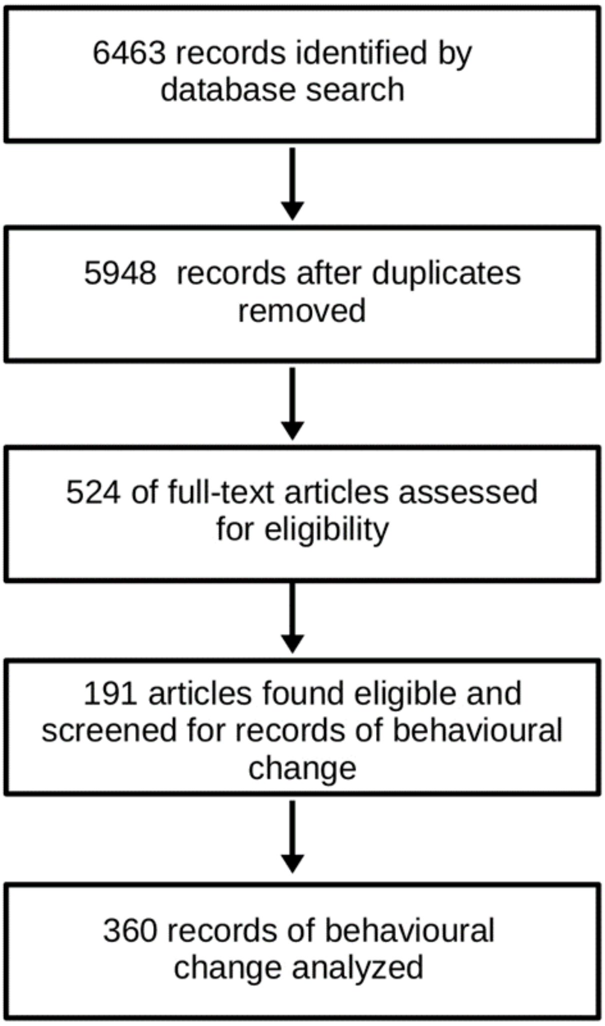

Figure S1.1: Flowchart with numbers of studies collected, scanned for eligibility and analysed in Ruland and Jeschke (2020), modified after Moher et al. (2014).

All types of behavioural change

Table S1: All combinations of taxonomic group, type of behavioural change, EEE questions, innovation questions and population trends. Total numbers of records are shown in the light blue row. For the population trend analyses, a subset of the data with robust information was used and therefore sample size is lower there. Percentages of records in each category are given in parentheses.

	amphibians	birds	mammals	defence	feeding	other
Type of behavioural change	17 total	50 total	19 total	38 total	38 total	10 total
defence	9 (53%)	17 (34%)	12 (63%)	N/A	N/A	N/A
feeding	3 (18%)	29 (58%)	6 (32%)	N/A	N/A	N/A
other	5 (29%)	4 (8%)	1 (5%)	N/A	N/A	N/A
Eco-evolutionary experience						
new guild	5 (29%)	5 (10%)	2 (11%)	8 (21%)	4 (11%)	0 (0%)
new functional trait	8 (47%)	25 (50%)	14 (74%)	31 (82%)	11 (29%)	5 (50%)
no new trait	9 (53%)	25 (50%)	5 (26%)	7 (18%)	27 (61%)	5 (50%)
Innovation						
rate change	17 (100%)	22 (44%)	10 (53%)	32 (84%)	12 (32%)	5 (50%)
object change	0 (0%)	28 (56%)	9 (47%)	6 (16%)	26 (68%)	5 (50%)
technical change (incl. obj.)	0 (0%)	3 (6%)	1 (5%)	2 (5%)	2 (5%)	0 (0%)
Population trend (subset)	9 total	37 total	14 total	30 total	27 total	3 total
population increase	0 (0%)	23 (62%)	4 (29%)	12 (40%)	15 (56%)	0 (0%)
stable population	4 (44%)	5 (14%)	5 (36%)	4 (13%)	7 (26%)	3 (100%)
population decline	5 (56%)	9 (24%)	5 (36%)	14 (47%)	5 (19%)	0 (0%)

Phylogenetic correction: each genus only considered once

The relationship between behavioural change and EEE remain qualitatively the same when each genus is only considered once. In particular, object changes are more commonly observed as EEE increases ($p < 0.05$, $X^2 = 12$, all following Chi-square tests are Pearson's Chi-square tests with 100'000 simulations, Fig. S1.2). To only consider each genus of non-native species once, we split all cases by behavioural change and EEE; within each of these subsets, each unique genus was considered as one case.

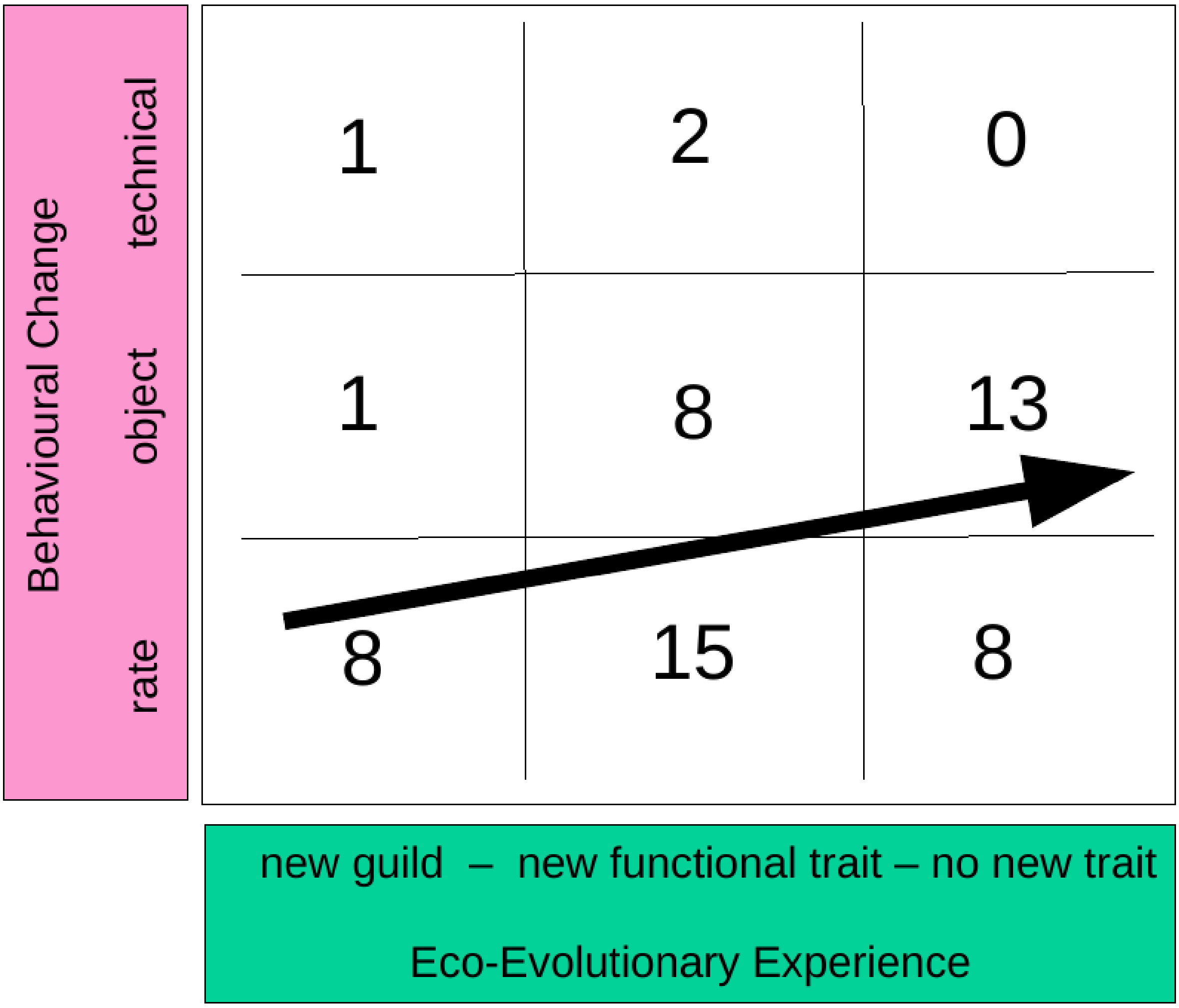

Figure S1.2: Records of a new guild and/or a new functional trait in the non-native species and the type of behavioural change in the native species (rate-, object- or technical change), with each genus only considered once. The level of eco-evolutionary experience (EEE) increases from left to right, the level of innovation from the bottom to the top. The arrow denotes a trend towards more object innovations with increasing EEE. Total n=56 records.

The results for population trends are also qualitatively the same when each genus is only considered once (Fig. S1.3). Population trends are more positive with increasing EEE but not significantly so. Object changes more often lead to a positive population trend than rate changes ($p < 0.05$, $X^2 = 6.7$). Rate changes are more common at lower EEE, and object changes are more common at high EEE.

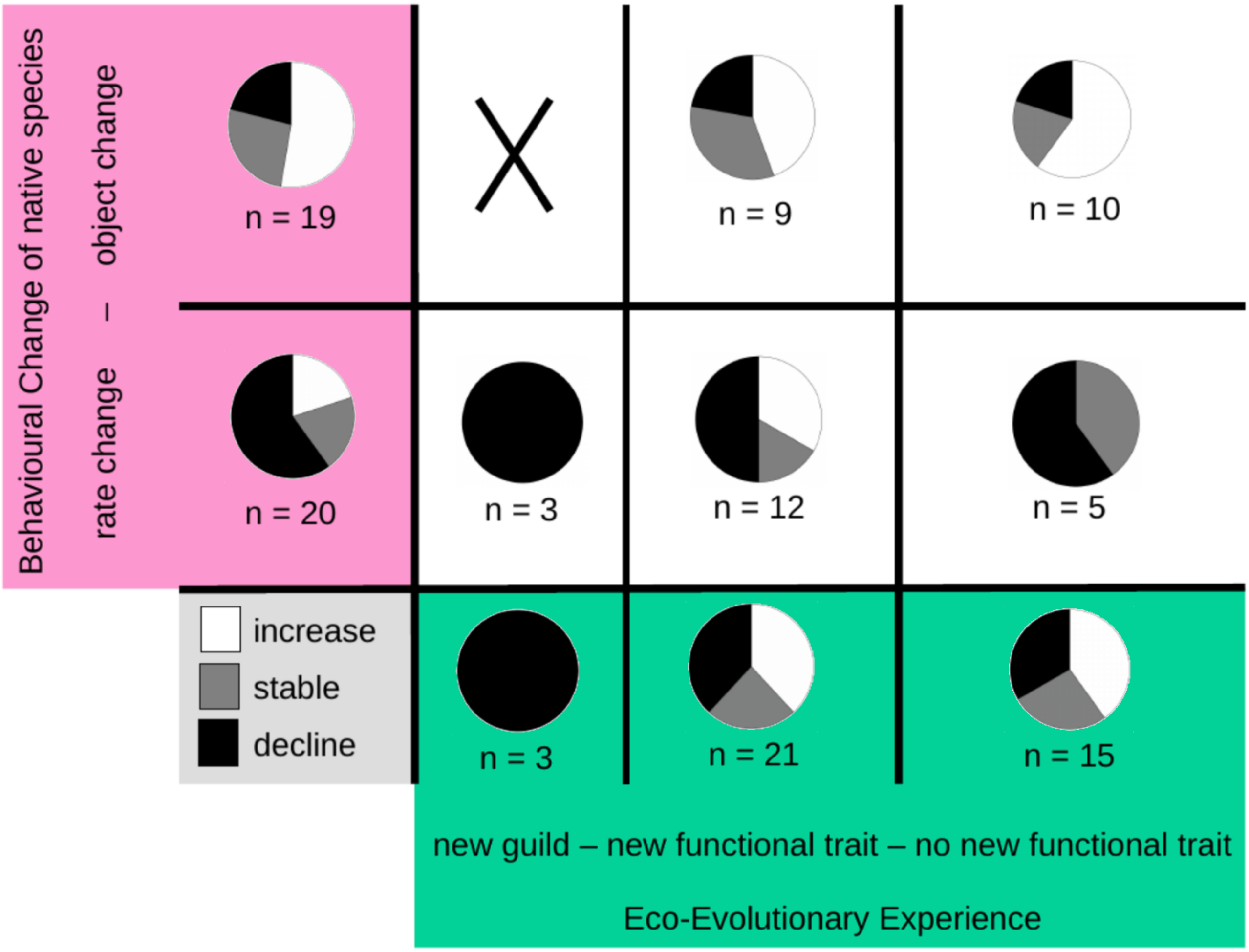

Figure S1.3: Post-invasion population trends of the focal native species (decline, stable or increase in the pie charts) depending on their eco-evolutionary experience (new guild or new functional trait, green boxes) and the degree of innovation in their behavioural change (rate change or object change, outcome in magenta boxes). Each genus is only considered once.

Taxonomically homogeneous subsample: Focus on native birds

When only considering behavioural changes of native bird species, and thus using a taxonomically more homogenous dataset, object changes resulted in generally more positive population trends than rate changes ($p < 0.05$, $X^2 = 7.8$). The same effect was observed within interactions with non-native species without new functional traits ($p < 0.05$, $X^2 = 9.9$) and with new functional traits, the latter not significantly so ($p = 0.32$, $X^2 = 2.6$). EEE alone did not have a significant effect on the population trend ($p = 0.47$, $X^2 = 1.7$).

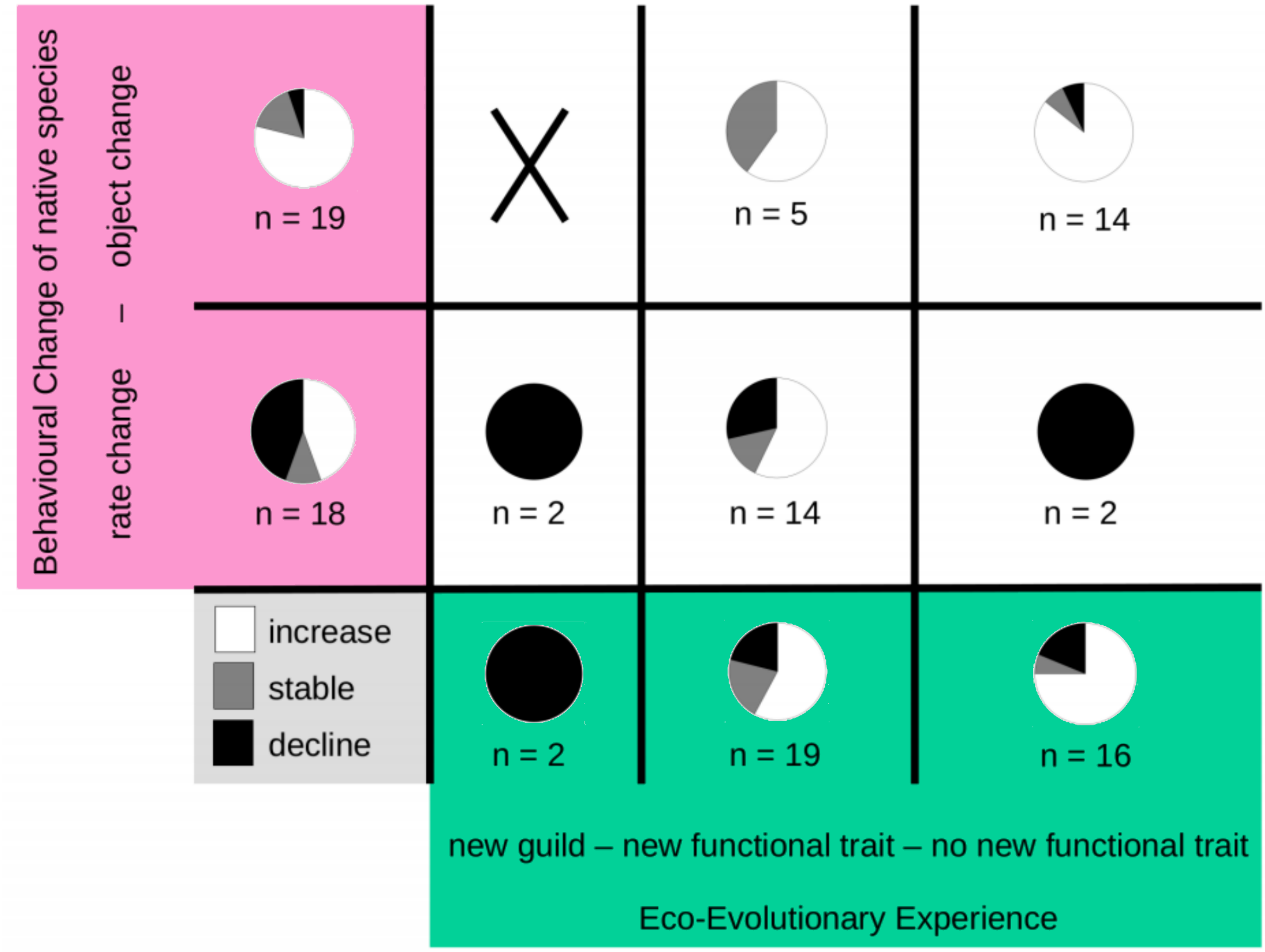

Figure S1.4: Post-invasion population trends of focal native bird species (decline, stable or increase in the pie charts) depending on their eco-evolutionary experience (new guild or new functional trait, green boxes) with a non-native species and the degree of innovation in their behavioural change (rate change or object change, outcome in magenta boxes).
